# Licochalcone A Suppresses the Proliferation of Osteosarcoma Cells through Autophagy and ATM-Chk2 Activation

**DOI:** 10.3390/molecules24132435

**Published:** 2019-07-02

**Authors:** Tai-Shan Shen, Yung-Ken Hsu, Yi-Fu Huang, Hsuan-Ying Chen, Cheng-Pu Hsieh, Chiu-Liang Chen

**Affiliations:** 1 Department of Orthopedic Surgery, Changhua Christian Hospital, Changhua 50006, Taiwan; 2 Orthopedics & Sports Medicine Laboratory, Changhua Christian Hospital, Changhua 50006, Taiwan; 3 Department of Nursing, Da Yeh University, Changhua 51591, Taiwan

**Keywords:** Licochalcone A, ATM-Chk2, autophagy, osteosarcoma

## Abstract

Licochalcone A, a flavonoid extracted from licorice root, has been shown to exhibit broad anti-inflammatory, anti-bacterial, anticancer, and antioxidative bioactivity. In this study, we investigated the antitumor activity of Licochalcone A against human osteosarcoma cell lines. The data showed that Licochalcone A significantly suppressed cell viability in MTT assay and colony formation assay in osteosarcoma cell lines. Exposure to Licochalcone A blocked cell cycle progression at the G2/M transition and induced extrinsic apoptotic pathway in osteosarcoma cell lines. Furthermore, we found the Licochalcone A exposure resulted in rapid ATM and Chk2 activation, and high levels of nuclear foci of phosphorylated Chk2 at Thr 68 site in osteosarcoma cell lines. In addition, Licochalcone A exposure significantly induced autophagy in osteosarcoma cell lines. When Licochalcone A-induced autophagy was blocked by the autophagy inhibitor chloroquine, the expression of activated caspase-3 and Annexin V positive cells were reduced, and cell viability was rescued in Licochalcone A-treated osteosarcoma cell lines. These data indicate that the activation of ATM-Chk2 checkpoint pathway and autophagy may contribute to Licochalcone A-induced anti-proliferating effect in osteosarcoma cell lines. Our findings display the possibility that Licochalcone A may serve as a potential therapeutic agent against osteosarcoma.

## 1. Introduction

Osteosarcoma is the most common primary tumor of bone. It is a highly malignant form of bone cancer characterized by osteoid production. Osteosarcoma arises predominantly in adolescents and children, with a second incidence peak in the elderly [1,2]. Osteosarcoma often originates from long bones including the distal femur, proximal tibia, and proximal humerus. It is characterized by high malignancy, frequent recurrence, and distant metastasis. By next-generation sequencing, several groups have revealed huge somatically mutated genes in osteosarcoma samples from patients. There are several cancer-causing genes showing high frequency of mutation in osteosarcoma samples including TP53, RB1, BRCA2, and DLG2 [3].

The major treatment for osteosarcoma is surgery. However, the survival rate of patients with osteosarcoma treated with surgery alone is about 15–17% [4]. In the early 1970s, chemotherapy was introduced as adjuvant treatment to facilitate surgical resection. The common chemotherapy protocols comprise of drugs namely, cisplatin, doxorubicin, and high-dose methotrexate. This incorporation has results in an overall 5-years survival rates that approach 70%. Unfortunately, 30% of patients diagnosed with osteosarcoma will not survive for more than 5 years. Treatment often fails due to the development of metastasis, chemo-resistance, and relapse of disease. A total of 30–40% of patients with localized osteosarcoma will develop a local or distant recurrence [5], resulting in only 23–29% overall 5 year survival rates in these patients [6].This outcome has remained virtually unchanged over the past 30 years. Therefore, novel strategies and effective drugs are urgently required, especially for patients suffering from advanced osteosarcoma.

**Figure 1:**
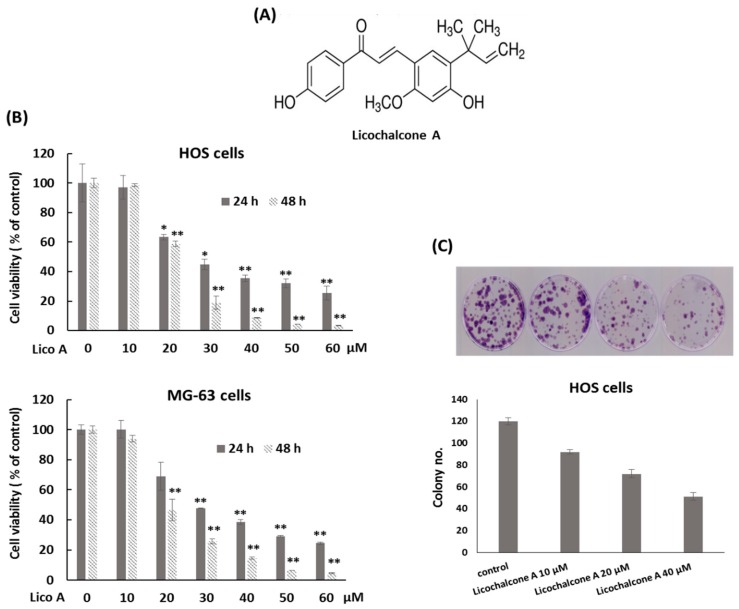
Licochalcone A inhibits cell viability of osteosarcoma. (**A**) The chemical structure of Licochalcone A. (**B**) Licochalcone A inhibits osteosarcoma cell growth in a dose-dependent manner. MTT assays were performed with osteosarcoma HOS and MG-63 cells exposed to Licochalcone A (Lico A) in the indicated concentrations. Experiments were conducted with three biological replicates per treatment, and the values represent the mean ± SD. (*) *p* < 0.01 and (**) *p* < 0.001 as compared with the untreated cells. (**C**) Licochalcone A suppresses colony formation of osteosarcoma cell lines. HOS cells were plated in colony formation assays after treatment with Licochalcone A for 7 h. Five hundred cells were plated per dish. All experiments were performed in triplicate, and the figure above shows a representative example.

Recent progress has focused on the chemotherapy by natural compounds for their anti-growth activity against cancer cells. These compounds may exhibit less adverse effects compared to synthetic chemicals [7,8]. Licorice (Glycyrrhiza glabra) is a well-known herb named for its unique sweet flavor. It is utilized to add flavor to foods, beverages, and tobacco, and is widely used as an herbal medicine. Licorice is used for gastritis, ulcers, cough, bronchitis, and inflammation [9]. Licochalcone A is an oxygenated chalcone (a type of natural phenol) (Figure 1A) that can be isolated from the roots of Glycyrrhiza species (such as *G. glabra*, *G. inflata*, and *G. eurycarpa*) which belong to the plant family of Fabaceae [10]. It has been demonstrated to possess antiviral [11] and antimicrobial activities [12]. In addition, literatures have shown that Licochalcone A has antioxidant [13], anti-angiogenesis [14], anti-inflammation [15], and anti-tumor effects [16]. Licochalcone A induces cell cycle arrest at S and G2/M phase and triggers intrinsic and extrinsic apoptosis in oral squamous cell carcinoma cells [17]. Licochalcone A suppresses the proliferation of lung cancer cell via G2/M cell cycle arrest and ER stress [18]. Licochalcone A inhibits PI3K/Akt/mTOR activation, and promotes autophagy and apoptosis in breast cancer cells [19] and cervical cancer cells [20]. Licochalcone A induces apoptotic cell death via p38 activation in human nasopharyngeal carcinoma cells [21] and head and neck squamous carcinoma cells [22]. In this study, we evaluated the potential anti-tumor effect of Licochalcone A against osteosarcoma.

## 2. Results

### 2.1. Licochalcone A Inhibits Osteosarcoma Cell Viability and Proliferation

Mutations in TP53 have been observed in 50–90% of osteosarcoma. It is most frequently mutated gene in osteosarcoma [3]. To mimic this genetic background in in vitro study, osteosarcoma HOS cells (R156P p53 mutation) [23] and MG-63 (mutant-p53, harboring a rearrangement in intron 1) [24,25] were used. Cell viability of osteosarcoma cell lines after exposure to various concentrations of Licochalcone A (0–60 μM) was detected by the MTT assay. The data showed that Licochalcone A clearly inhibited cell viability of osteosarcoma HOS cells and MG-63 cells at the concentrations of 20–60 μM following exposure for 24 h and 48 h compared with the control group (Figure 1B). The half maximal inhibitory concentration (IC_50_) calculated based on data of the MTT assays for HOS cells were 29.43 μM at 24 h and 22.48 μM at 48 h, and those for MG-63 cells were 31.16 μM at 24 h and 22.39 μM at 48 h. Next, the colony formation assay was performed to examine the effect of Licochalcone A on cell proliferating capacity. The results showed that the treatment with Licochalcone A reduced colony number at the concentrations of 10–40 μM compared with the control group in osteosarcomas HOS cells (Figure 1C). These data indicate that Licochalcone A significantly inhibits the cell viability of osteosarcoma cell lines in a dose-dependent manner.

### 2.2. Licochalcone A Induces Apoptosis and Cell Arrest

**Figure 2:**
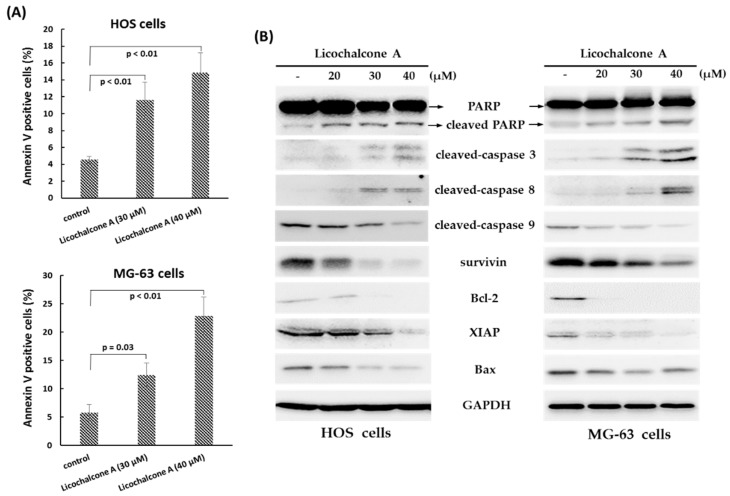
Licochalcone A induces apoptosis in osteosarcoma cells. Osteosarcoma HOS cells or MG-63 cells were treated with Licochalcone A (30 μM) for 24 h. To detect apoptosis, the HOS cells or MG-63 cells were stained with Annexin V and propidium iodide (PI), and analyzed using flow cytometry. Quantitative results of Annexin V positive cells are shown (**A**). Expression of apoptosis-related proteins was measured by Western blotting (**B**). Experiments were conducted with three biological replicates per condition, and the values represent the mean ± SD.

To determine whether programmed cell death was involved in the anti-proliferative effect of Licochalcone A, we analyzed the rate of apoptosis cells in Licochalcone A-treated HOS cells and MG-63 cells by Annexin V and PI staining observed by flow cytometry. The data showed that the rate of Annexin V positive cells was significantly increased after exposure to Licochalcone A (30 μM or 40 μM) for 24 h in both lines of osteosarcoma cells (Figure 2A), indicating Licochalcone A has the potential to induce apoptosis in osteosarcoma cell lines. To determine whether caspase activation was involved in Licochalcone A–induced apoptosis, we measured the protein levels of the activated forms of caspase-3, -8, and -9 and PARP by Western blot analysis in treated HOS cells and MG-63 cells. The data showed that treatment with Licochalcone A (20–40 μM) for 24 h resulted in up-regulated activated forms of caspase 8, caspase 3, and PARP, but decreased activated forms of caspase 9 and Bax (Figure 2B). Besides, we also observed that treatment with Licochalcone A both resulted in down-regulation of pro-survival protein Bcl-2 and inhibitors of the apoptosis protein (IAP) family such as XIAP and survivin (Figure 2B). These findings suggest that Licochalcone A induces apoptosis by caspase 8 and caspase 3 signaling pathway.

**Figure 3:**
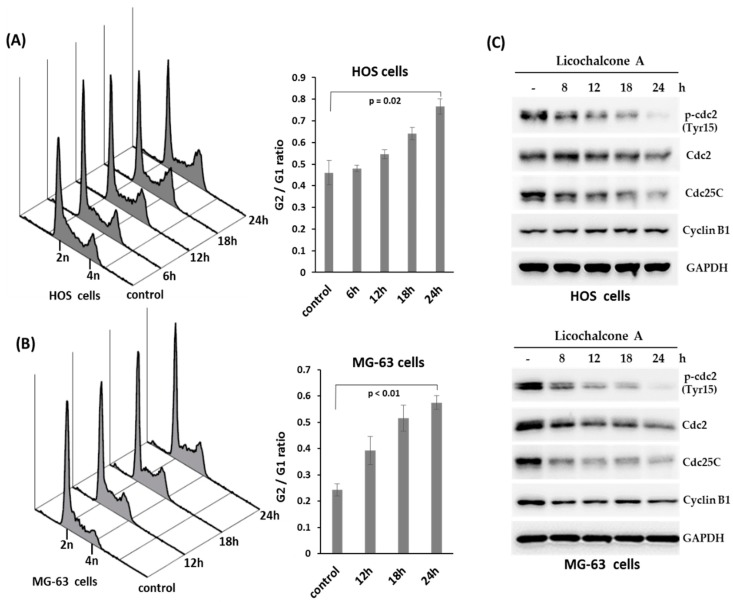
Licochalcone A induces G2/M phase arrest in osteosarcoma cell lines. HOS cells (**A**) or MG-63 cells (**B**) were treated with Licochalcone A (30 μM), and harvested in the indicated time points. The cells were stained with propidium iodide and analyzed by flow cytometer. 2n corresponds to G1 phase cells and 4n corresponds to the G2/M phase cells. The ration of G2/M to G1 phase cells were showed in right panel. Experiments were conducted with three biological replicates per condition, and the values represent the mean ± SD. (**C**) Licochalcone A decreases the expression of p-cdc2, cdc2, and cdc25c in osteosarcoma cell lines. HOS cells or MG-63 cells were treated with Licochalcone A (30 μM), and harvested in the indicated time points. The treated cells were analyzed by Western blotting using the indicated antibodies.

Cell cycle distribution of HOS cells or MG-63 cells treated with Licochalcone A (30 μM, a concentration close to IC_50_ at 24 h for both cell lines) for different time points were analyzed by flow cytometry. The results showed that a significant accumulation of 4N cells (G2/M phase cells) was induced in Licochalcone A-treated HOS cells (Figure 3A) and MG-63 cells (Figure 3B). Furthermore, we evaluated the expression of proteins that regulate the G2/M phase transition by Western blot assay. The data showed that the protein level of phospho-cdc2, cdc2, and Cdc25C were decreased in treated both cell lines, but Cyclin B1 protein levels had no apparent change in HOS cells and was decreased in MG-63 cells (Figure 3C), indicating Licochalcone A induces G2/M phase arrest of osteosarcoma cell lines.

### 2.3. Activation of Chk2 and ATM in Response to Licochalcone A

**Figure 4:**
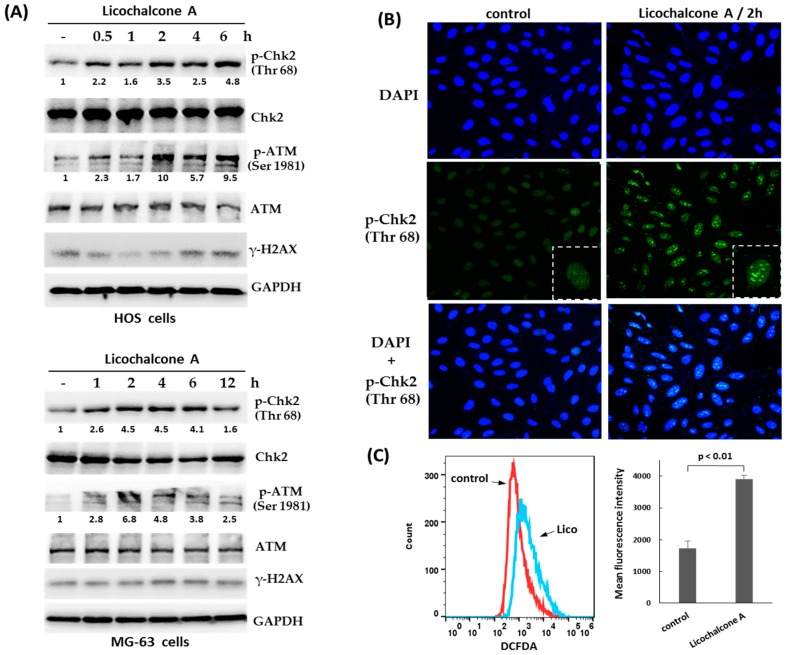
Activation of Chk2 and ATM in response to Licochalcone A. (**A**) ATM-Chk2 pathway is activated in Licochalcone A -treated HOS cells. HOS cells or MG-63 cells were treated with Licochalcone A (30 μM), and harvested in the indicated time points. The treated cells were analyzed by Western blotting using the indicated antibodies. The levels of p-Chk2 (Thr 68) and p-ATM (Ser 1981) were quantified and are shown below each blot. (**B**) Licochalcone A induces phospho-Chk2 T68 foci. HOS cells were treated with Licochalcone A (30 μM), and 2 h later were fixed with formaldehyde, permealized with Triton X-100, and then immunostained with antibody to phospho-Chk2 T68 (green color) and 4′,6-diamidino-2-phenylindole (DAPI) for labeling nucleus (blue color). (**C**) Licochalcone A enhances reactive oxygen species (ROS) generation. MG-63 cells were treated with Licochalcone A (Lico) for 2 h. Intracellular ROS was analyzed by flow cytometry using 2′,7′-dichlorofluorescin diacetate (DCFDA) staining and is shown as median fluorescence intensity. Mean ± SD. is plotted for 3 replicates from each condition.

To identify the mechanism involving in Licochalcone A-induced G2/M phase arrest in osteosarcoma cells, we tested the activation of ATM-Chk2 and ATR-Chk1, the primary pathway for activating G2/M checkpoint. The data showed that the treatment with Licochalcone A induced rapid phosphorylation of Chk2 (at threonine 68) and ATM (at serine 1981) (Figure 4A), but did not induce Chk1 phosphorylation (at serine 345) (data not shown). Interestingly, we observed the threonine 68-phosphorylated form of Chk2 formed distinct nuclear foci in response to Licochalcone A treatment in HOS cells (Figure 4B). These data suggest that ATM-Chk2 pathway may contribute to Licochalcone A-induced G2/M phase arrest in osteosarcoma cells. Recently, it has been reported that oxidative stress can activate ATM [26]. We examined the cellular redox status following Licochalcone A treatment in osteosarcoma MG-63 cells using 2′,7′-dichlorofluorescin diacetate (DCFDA), a fluorogenic dye that measures reactive oxygen species (ROS) within the cell. The data showed that ROS levels were significantly elevated in Licochalcone A-treated cells. Thus, we propose oxidative stress to be involved in Licochalcone A-mediated activation of ATM.

### 2.4. Autophagy is Involved in Licochalcone A—Induced Apoptosis

**Figure 5:**
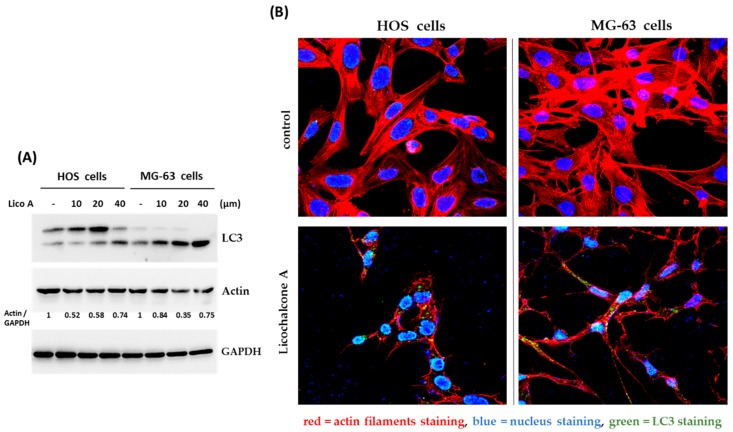
Autophagy is induced in Licochalcone A -treated osteosarcoma cells. HOS cells and MG-63 cells were treated with indicated concentrations of Licochalcone A (Lico A) for 24 h. The treated cells were analyzed by Western blotting using the indicated antibodies (**A**), or were immunostained with LC3 antibody for autophagy formation (green color), phalloidin-iFluor 594 reagent for labeling actin filaments (red color), and 4′,6-diamidino-2-phenylindole (DAPI) for labeling nucleus (blue color) (**B**). The levels of actin were quantified and are shown below the blot in Western blotting.

**Figure 6:**
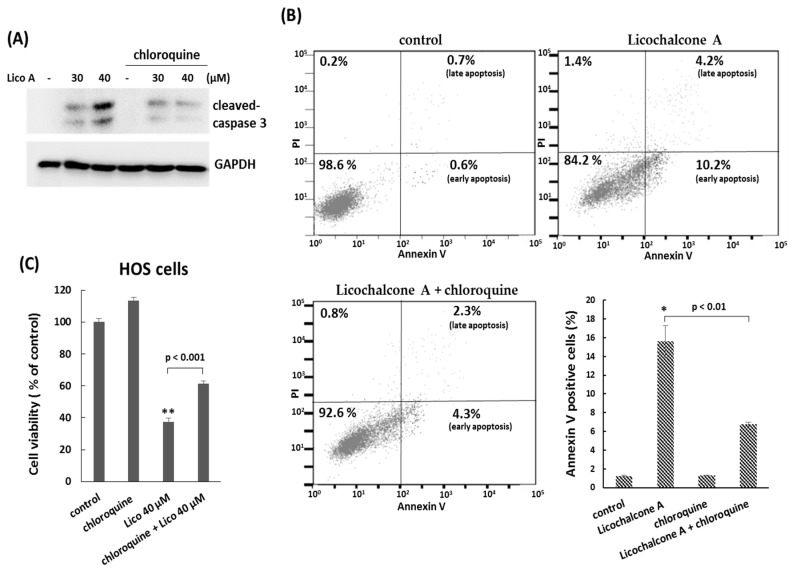
Autophagy is involved in the apoptosis effect of Licochalcone A treatment. (**A**) Autophagy inhibitor, chloroquine, suppresses Licochalcone-induced caspase 3 activation. HOS cells were treated with indicated concentrations of Licochalcone A (Lico A) with or without chloroquine for 24 h. The treated cells were analyzed by Western blotting using the indicated antibodies. (**B**) Autophagy inhibitor chloroquine suppresses Licochalcone-induced apoptosis. HOS cells were treated with Licochalcone A (Lico A) (40 μM) with or without chloroquine for 24 h. To detect apoptosis, the HOS cells were stained with Annexin V and propidium iodide (PI), and analyzed using flow cytometry. Quantitative results of Annexin V positive cells are shown in the lower panels. (**C**) Autophagy inhibitor chloroquine rescues the anti-proliferative effect of Licochalcone A treatment. HOS cells were treated as described in (**B**). MTT assay was performed to determine cell viability in treated cells. Experiments were conducted with three biological replicates per treatment, and the values represent the mean ± SD. (*) *p* < 0.01 and (**) *p* < 0.001 as compared with the control group.

Autophagy is a cellular process used to recycle or degrade proteins and cytoplasmic organelles in response to stress. Accumulating evidence has revealed that a large number of natural compounds are involved in autophagy modulation, either inducing or inhibiting autophagy [27]. Therefore, we decided to demonstrate the interaction between Licochalcone A and autophagy. First, we analyzed the formation of LC3A/B-II, the marker protein for autophagosomes by Western blotting assay. The data showed that the protein level of LC3A/B-II was significantly induced by Licochalcone A exposure in osteosarcoma HOS cells and MG-63 cells (Figure 5A), suggesting Licochalcone A has potential to induced autophagy. This result was further confirmed by LC3 puncta formation assay using confocal microscopy (Figure 5B). In addition, the marked reduction of actin filaments was observed in Licochalcone A-treated cells (Figure 5B). Next, we examined the role of autophagy in Licochalcone A-treated osteosarcoma cells. Once the autophagy was blocked by autophagy inhibitors, chloroquine, the cleaved caspase 3 and Annexin V positive cells were reduced (Figure 6A,B), and cell viability was rescued (Figure 6C) in Licochalcone A-treated HOS cells, indicating that the autophagy is associated with Licochalcone A-induced apoptosis in osteosarcoma HOS cells.

## 3. Discussion

In this study, we demonstrated that Licochalcone A suppressed cell viability through the induction of cell cycle arrest at G2/M phase and caspase 8/3-dependent apoptosis in osteosarcoma cell lines. Our data further indicated that the activation of ATM/Chk2 and autophagy may be involved in Licochalcone A-induced anti-proliferating effect in osteosarcoma cell lines.

Apoptosis can be conducted in two major signaling pathways: The extrinsic pathways and the intrinsic pathways. The extrinsic pathways involve transmembrane receptor-mediated interactions, and are modulated by caspase 8 and caspase 3. The intrinsic pathways involve mitochondrial, and are controlled by several proteins including Bax, Bcl-2 protein family, cytochrome c, caspase 9, and caspase 3 [28,29]. In this report, we showed that Licochalcone A treatment induced cleaved-caspase 8 and caspase 3, but decreased the levels of Bax and cleaved-caspase 9, indicating that Licochalcone A triggers apoptosis that mediated by the extrinsic pathways in osteosarcoma cell lines. However, several literatures report that caspase 9-mediated intrinsic pathways could be induced by Licochalcone A in other cell types such as glioma stem cells [16], and nasopharyngeal carcinoma cells [21].

In response to DNA damage, ATM/Chk2 and ATR/Chk1 pathways have the central roles in maintaining genome stability by inducing cell cycle arrest, apoptosis, and DNA repair [30]. As shown in Figure 4A, the activation of ATM and Chk2 could be observed as early as 0.5–1 h after Licochalcone A treatment, whereas the level of γH2AX, the DNA double-strand breaks biomarker, didn’t be significantly elevated. Therefore, we propose that Licochalcone A-mediated activation of ATM/Chk2 is unlikely due to direct damage of DNA. However, it was still uncertain how Licochalcone A activated ATM/Chk2 pathways. Recently, it has been reported oxidative stress such as H_2_O_2_ treatment can activate ATM-Chk2 in the absence of DNA double-strand breaks [31]. Our data showed that reactive oxygen species (ROS) levels were significantly elevated in Licochalcone A-treated cells. However, the role of oxidative stress in Licochalcone A-mediated activation of ATM-Chk2 should be further confirmed in the future.

Autophagy is an important catabolic process used to degrade or recycle proteins and cytoplasmic organelles in response to stress. Nevertheless, the cellular outcome for inducing autophagy is different and depends on the context of cells [32,33,34]. The literature shows that Licochalcone A induces autophagy in cervical cancer cells, and treatment with autophagy inhibitors enhances Licochalcone A-induced apoptosis in these cells [20]. However, in osteosarcoma cell lines, we showed Licochalcone A-induced apoptosis was suppressed, and cell viability was rescued as the induced autophagy was blocked by autophagy inhibitors. We suggest Licochalcone A-induced autophagy has the positive effect in promoting apoptosis in osteosarcoma cell lines. In addition, reactive oxygen species (ROS) have been shown to be a general inducer of autophagy [35]. It is possible that the elevated ROS by Licochalcone A treatment may contribute to induce autophagy formation in osteosarcoma cell lines.

In conclusion, the present study demonstrated that Licochalcone A exhibits antitumor activity in vitro by inhibiting cell viability, arresting cell cycle progression and inducing apoptosis in osteosarcoma cells. It has been reported that Licochalcone A has less cytotoxic effect on normal cells (HK-2 and WI-38) [20]. Therefore, Licochalcone A may serve as a potential therapeutic agent against osteosarcoma

## 4. Materials and Methods

### 4.1. Cell Culture and Chemicals

Human HOS and MG-63 osteosarcoma cells were purchased from the Bioresource Collection and Research Center (Hsinchu, Taiwan). HOS and MG-63 cells were maintained in Minimum Essential Medium (#11095-080; Gibco, Carlsbad, CA, USA) supplemented with 10% fetal bovine serum (Gibco, Carlsbad, CA, USA), 1% penicillin/streptomycin (Gibco, Carlsbad, CA, USA). PCR mycoplasma detection kit (BSMP-101, Bio-Smart, Hsinchu, Taiwan) was used to detect mycoplasma infection every three months. The used cells are mycoplasma-negative. Licochalcone A were obtained as a power from Santa Cruz Biotechnology (sc-319884). The purity of Licochalcone A was more than 96%.

### 4.2. Colony Formation Assay

HOS cells were briefly treated with Licochalcone A at various concentrations (0, 10, 20, and 40 μM). Once the expression of proteins that regulate the G2/M phase transition such as Cdc25C were decreased, starting at 6–8 h after Licochalcone A treatment (Figure 3C), the treated cells were washed by PBS three times, trypsinized, plated and maintained onto 35 mm dishes (500 cells/dish) with drug-free complete medium and cultured for another 10–14 days to allow colony formation. Colonies were fixed in 70% ethanol and stained by 1% crystal violet solution before counting.

### 4.3. Cell Viability Assay

The cytotoxic activity of Licochalcone A was tested using the MTT assay. The human osteosarcoma HOS and MG-63 cells were seeded in 24-well plates for overnight. The cells were treated with different concentrations of Licochalcone A for 24 h or 48 h. At the end of the assay time, the cells was incubated with 15 µL of MTT solution (5 mg/mL) (Invitrogen, Carlsbad, CA, USA) for 2 h at 37 °C. After removing the cultured medium, 200 µL of dimethyl sulfoxide (DMSO) was added to each well. Absorbance at 590 nm of the dissolved formazan product was read on an automated microplate spectrophotometer (Thermo Multiskan SPECTRUM, Thermo Fisher Scientific, Waltham, MA, USA). The half maximal inhibitory concentration (IC_50_) for HOS and MG-63 cells were calculated by the “Forecast” function in Microsoft Excel.

### 4.4. Cell Cycle Analysis

The cells were washed by PBS two times, trypsinized, fixed with ice-cold 100% ethanol and kept on −20 °C for overnight. Cells were rehydrated with cold PBS, and then resuspended in PBS with propium iodine (40 µg/mL) (#P4170; Sigma-Aldrich, St. Louis, MO, USA) and Ribonuclease A (0.2 µg/mL) at room temperature for 30 min in the dark. The content of DNA in each sample were analyzed by a Cytomics^TM^ FC500 flow cytometer (Beckman Coulter; Brea, CA, USA).

### 4.5. Apoptosis Assay

Licochalcone A-induced apoptosis in HOS cells was determined by flow cytometry using the Annexin-V-FITC staining kit (Becton Dickinson, San Jose, CA, USA) according to the manufacturer’s protocol. Briefly, the treated cells were trypsinized, and washed twice by cold PBS. The cells were incubated with 100 μL of 1× binding buffer with 5 μL of FITC Annexin V and 5 μL of propidium iodide for 15 min at room temperature (RT) in the dark. After incubation, 400 μL of 1× binding buffer was added to each tube, and the fluorescence was measured by a Cytomics^TM^ FC500 flow cytometer (Beckman Coulter, Miami, FL, USA).

### 4.6. Immunofluorescence Assays

For immunofluorescence analysis, cells were grown on glass coverslips and fixed in a 4% formaldehyde solution for 15 min at room temperature. After rinsing three times with PBS for 5 min each, the cells were blocked in blocking buffer (1XPBS, 5% normal serum, 0.3% Triton X-100) for 60 min at room temperature. After removing blocking solution, the primary antibodies were diluted in antibody dilution buffer (1XPBS, 1% BSA, 0.3% Triton X-100), and incubated on cells overnight at 4 °C. The coverslips were washed with PBS three times and incubated in fluorescent-dye conjugated secondary antibody diluted in antibody dilution buffer for 2 h at room temperature. The cells were washed with PBS three times and counterstained with DAPI. The cells were examined and photographed by immunofluorescence microscopy. The primary antibodies was used in this assay included Phospho-Chk2 (Thr68) (#2197; Cell Signaling, Danvers, MA, USA), LC3A/B (#12741; Cell Signaling, Danvers, MA, USA). Phalloidin-iFluor 594 reagent (ab176757; Abcam, Cambridge, UK) were used for labeling actin filaments

### 4.7. Western Blot Analysis

Whole cell lysates were produced using TEGN buffer (10 mM Tris, pH 7.5, 1 mM EDTA, 420 mM NaCl, 10% glycerol, and 0.5% Nonidet P-40) containing proteases inhibitor cocktail (Roche, Mannheim, Germany), phosphatase inhibitors (Roche, Mannheim, Germany), and 1 mM dithiothreitol (DTT). For Western blotting, the cell lysates were boiled in protein sample buffer (2 Mβ-mercaptoethanol, 12% sodium dodecyl sulfate (SDS), 0.5 M Tris, pH 6.8, 0.5 mg/mL bromophenol blue, and 30% glycerol). The samples were analyzed by 8–12% SDS-polyacrylamide gel electrophoresis (PAGE). The primary antibodies were listed as following: cleaved caspase-3 (#9661; Cell Signaling, Danvers, MA, USA), cleaved caspase-8 (#9496; Cell Signaling, Danvers, MA, USA), cleaved caspase-9 (#9508; Cell Signaling, Danvers, MA, USA), Bcl-2 (#9258; Cell Signaling, Danvers, MA, USA), XIAP (#2042; Cell Signaling, Danvers, MA, USA), Survivin (#2808; Cell Signaling, Danvers, MA, USA), GAPDH (#2118; Cell Signaling, Danvers, MA, USA), actin (A2066; Sigma-Aldrich, St. Louis, MO, USA), PARP (#9542; Cell Signaling, Danvers, MA, USA), Bax (#2772; Cell Signaling, Danvers, MA, USA), Phospho-cdc2 (Tyr15) (#4539; Cell Signaling, Danvers, MA, USA), Phospho-Chk2 (Thr68) (#2197; Cell Signaling, Danvers, MA, USA), LC3A/B (#12741; Cell Signaling, Danvers, MA, USA), cdc2 (06-923SP; Millipore, Temecula, CA, USA), p-ATM (Ser1981) (sc-47739; Santa Cruz, CA, USA), ATM (GTX70103; GeneTex, Irvine, CA, USA), Chk2 (#05-649; EMD Millipore, Temecula, CA, USA).

### 4.8. Detection of Intracellular ROS

Cellular ROS levels were measured in live cells by 2′,7′-dichlorofluorescin diacetate (DCFDA) (#ab113851, Abcam, Cambridge, UK). After treatment with Licochalcone A, the cells were washed with serum free media, and incubate at 37 °C incubator for 30min with 10 μM DCFDA in serum free media. Finally, the cells were trypsinized and suspended in phosphate-buffered saline (PBS). The fluorescence was detected by a Cytomics™ FC500 flow cytometer (Beckman Coulter, Miami, FL, USA).

### 4.9. Statistical Analysis

The experimental data are expressed as mean ± standard deviation. Statistical differences between groups were conducted using the Student’s t-test. A *p*-value less than 0.05 was considered to indicate a statistically significant difference. All statistical analyses were performed using the software package GraphPad Prism (Version 4.0, GraphPad Software; San Diego, CA, USA).
